# Stability-indicating UPLC assay coupled with mass spectrometry for the analysis of vilanterol degradation products in human urine

**DOI:** 10.1038/s41598-024-52664-6

**Published:** 2024-01-30

**Authors:** Mohamed Tarek, Hebatallah A. Wagdy, Maha A. Hegazy, Nermine S. Ghoniem

**Affiliations:** 1 Pharmaceutical Chemistry Department, Faculty of Pharmacy, The British University in Egypt (BUE), Cairo, Egypt; 2 Health Research Center of Excellence, Drug Research and Development Group, Faculty of Pharmacy, The British University in Egypt, Cairo, Egypt; 3 Analytical Chemistry Department, Faculty of Pharmacy, Cairo University, Kasr-El Aini Street, Cairo, 11562 Egypt

**Keywords:** Analytical chemistry, Mass spectrometry

## Abstract

Vilanterol is a once-daily dose inhaler prescribed for asthma and chronic obstructive pulmonary disease. This study involved an investigation of vilanterol stability under acidic, basic, oxidative, thermal, and photolytic stress conditions. UPLC method was developed and validated for the analysis of vilanterol with its degradants. The drug was stable under photolytic and thermal stress conditions and degraded under acidic, basic, and oxidative stress conditions. Degradation kinetics was performed for acidic, basic and oxidative stress conditions. Kinetics parameters, K, half-life time (t_1/2_) and shelf-life time (t_90_) were assessed, and the degradation followed first order reaction. The method was linear from 0.10 to 100.00 µg mL^−1^ with accuracy, inter-day and intra-day precision from 99.45 to 100.02%, 0.391–0.694 and 0.041–0.345, respectively. Mass spectrometry was employed to elucidate the structure of the degradants, and the results revealed that certain degradation products were comparable to vilanterol metabolites. The World Anti-Doping Agency has prohibited the presence of vilanterol and its metabolites in athletes’ urine except for exercise bronchoconstriction with limited dose. So, quantification of vilanterol in the presence of its degradants was performed in human urine. The results revealed that the method was linear in range of 1.00 to 100.00 µg mL^−1^. Samples collection and experimental protocol was performed according to the guidelines of the Research Ethics Committee of the Faculty of Pharmacy, the British University in Egypt with approval No. CH-2305.

## Introduction

**Figure 1:**
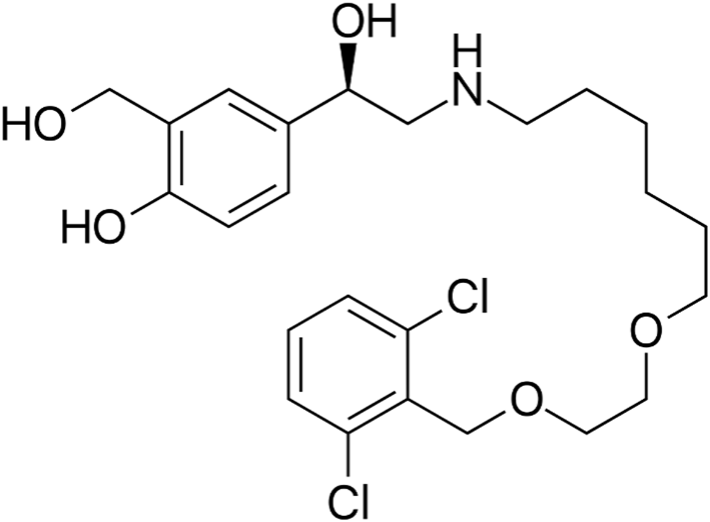
Chemical structure of vilanterol.

Vilanterol, (4-(2-((6-(2-((2,6-dichlorobenzyl)oxy)ethoxy)hexyl)amino)-1-hydroxyethyl)-2-(hydroxymethyl)phenol) Fig. 1 is an inhaled long-acting β_2_ agonist (LABA)^1^. It is a once-daily dose inhaler used for the management of asthma and chronic obstructive pulmonary disease (COPD)^1^. Asthma and COPD are long-term respiratory conditions that place substantial strain on healthcare systems globally. The WHO has projected that by 2030, COPD will be the third cause of death worldwide^2^. Over 300 million individuals worldwide, including approximately 15 million children, are affected by asthma. A projected 397,000 fatalities per year are attributed to this illness and COPD, which together account for a large portion of the world’s rising mortality rates^3,4^. Exacerbations are important events that can cause a quick decline in lung function, worsening general health, and an increased risk of death. A year after being admitted to the hospital owing to an exacerbation, the mortality rate may exceed 20%^5,6^. Limitations in airflow, hyperresponsiveness of the bronchi and inflammation are the main features of asthma and COPD. These features involve the activation of various inflammatory cells and mediators such cytokines, histamine, and leukotrienes, which results in a reversible restriction of airflow. Due to exposure to potentially dangerous substances such cigarette smoke, air pollution, stress, and genetic factors^7,8^.

Preventing deterioration in respiratory function, improving symptoms, avoiding exacerbations, and preventing pulmonary and extrapulmonary consequences are the main goals of treating asthma and COPD^9^.

In the control of COPD and asthma, β_2_ receptor agonists are crucial as they relax the airways, increasing airflow to the lungs. This could be achieved by acting directly on the β_2_ receptor. Stimulation of the β_2_ adrenergic receptor activates G-protein which stimulates adenylyl cyclase to elevate the levels of intracellular cyclic adenosine monophosphate. As a result, protein kinase A is stimulated, leading to the relaxation of the smooth muscles^10^.

Since the discovery of the short-acting β_2_ agonists with slow onset and short duration of action, continued research has proceeded to discover molecules with rapid onset and long duration of action that provide better control of symptoms. Consequently, LABA was developed as a twice-daily dose, which is more effective and convenient than short-acting bronchodilators in the treatment of severe COPD and asthma symptoms^8^. Furthermore, a once-daily dose LABA was developed for greater compliance and adherence to the treatment. They can be prescribed either alone or in combination with inhaled corticosteroids and/or muscarinic antagonists for the management of severe asthma and COPD symptoms^11,12^. Phenylethanolamines are the primary type of β_2_ agonists, which may have different substitutions on the aryl moiety and the terminal amino group and they are characterized by their lipophilicity^13^. The physicochemical characteristics of these compounds directly influence their metabolism^13,14^. Where, lipophilic drugs like vilanterol tend to undergo biotransformation reactions such as oxidation, hydroxylation, or dealkylation and conjugation to glucuronides or sulfates to produce more water-soluble derivatives. These derivatives are primarily excreted in the urine^14^. The World Anti-Doping Agency (WADA) releases a yearly list of banned substances, which includes β_2_ agonist drugs^15^. In particular, athletes are prohibited from using β_2_ agonists as they function as stimulants and can serve as anabolic agents at elevated doses. However, there are some β_2_ agonists allowed for use in exercise-induced bronchoconstriction within certain dosage limits^15–17^.

WADA was established to promote consistency in anti-doping policies across the globe and balance the effort to combat doping across different sports. WADA creates a structure for coordinated policies, regulations, and rules among sports organizations and public authorities. Additionally, it releases a yearly list of banned substances and methods that is consistent across different sports, with only a few exceptions^15^.

One of the β_2_ agonists that are banned for athletes to use is vilanterol due to its performance enhancing action and its anabolic effect^16^. It has high selectivity to β_2_ adrenergic receptors, prompting a favorable safety profile, 5 min onset with 24 h duration of action. The optimum therapeutic dose of vilanterol is 25.0 µg administrated via the inhalation route. The maximum drug concentration in plasma was reached after 0.5 h then it was metabolized mainly via de-alkylation and oxidation^14^. Vilanterol and its related materials were eliminated mainly in urine; about 70%; while the rest were eliminated through the fecal route^17,19^. Consequently, it is prohibited to detect vilanterol or its metabolite during the screening of doping agents in athletes^20^.

Conducting forced degradation and degradation kinetics investigations is crucial for determining the stability of the active pharmaceutical ingredient, drug safety, storage conditions, and shelf-life duration. Forced degradation entails subjecting the drug to more severe conditions compared to accelerated degradation studies, aimed at ensuring the drug’s quality and safety^21,22^.

Literature has witnessed a few analytical methods for the analysis of vilanterol using high-performance liquid chromatography (HPLC)^23–25^, spectrofluorimetric method^24^, stability indicating assay methods using HPLC^26–28^, and HPLC methods using mass detection for the screening of doping agents in athletes’ urine^17,29^.

As far as we can tell, there is only stability indicating assay methods using HPLC for vilanterol in literature^26–28^, but no one has been reported using ultra-performance liquid chromatography (UPLC) for the analysis of vilanterol in presence of its degradation products and no reported method showed the structural elucidation and the degradation pathway of the degradants. Therefore, the objective of this study is to develop and validate an accurate UPLC method for the precise and selective analysis of vilanterol. In addition, forced degradation and kinetic studies were carried out for vilanterol using the proposed UPLC method. Furthermore, mass spectrometry was employed for structure elucidation of the degradation products, and the degradation pathway as well. The proposed method was also applied for the analysis of vilanterol in the presence of its acidic, basic and oxidative degradation product in human urine samples. This method is promising, fast and feasible for the analysis of vilanterol and its metabolites in the doping tests of athletes.

## Materials and methods

### Chemicals and reagents

Vilanterol standard material was provided by Sigma-Aldrich (Steinheim, Germany) with certified purity of 99.95 ± 0.04%. HPLC grade acetonitrile and methanol and analytical grade ortho-phosphoric acid were provided by Fisher Scientific (Loughborough, Leicestershire, United Kingdom). Analytical grade sodium hydroxide (NaOH) pellets, hydrochloric acid (HCl) and hydrogen peroxide (H_2_O_2_) 30.0% (*v*/*v*) were obtained from Piochem (Cairo, Egypt). Human urine samples were collected from fasting healthy volunteers. Samples collection and experimental protocol was performed according to the guidelines of the Research Ethics Committee of the Faculty of Pharmacy, the British University in Egypt with approval No. CH-2305. The ethics committee was registered and recognized by the Egyptian Network of Research Ethics Committees (ENREC).

### Instruments

A Thermo Fisher UHPLC Dionex UltiMate 3000 (Germering, Germany) was used. It is composed of pump (ISO 3100SD), auto-sampler (WPS-3000SL), column oven (TCC-3000 SD) and diode array detector (DAD 3000RS) (Germering, Germany). The software was Chromeleon of version 6.8 (Germering, Germany). The pH was adjusted, and samples were neutralized using (Jenway pH meter, 3310, Dunmow, Essex, UK). De-ionized water was generated by a purification system (Thermo Scientific, Barnstead Smart2Pure 3-UV, Hungary). A water bath (Wisd – WSB-18, Berlin, Germany) was used for adjusting the temperature in the forced degradation study. An oven (Binder, Germany) was used for the thermal stability test of the drug. The photolytic stability of the drug was assessed using, UV lamp with power of 8 W (UVP-Upland, USA). Waters Acquity Triple quadrupole mass spectrometer (USA) with electrospray ionization (ESI) mode (Waters Corp., Milford, MA) operated with software mass lynx V4.1 was used for the structure elucidation of the degradants. Drug extraction from urine samples was achieved using ultra-sonicator (Elmasonic S 60 (H), Germany). The vortex of urine samples was done by (VELP Scientifica, Europe) and the centrifugation was performed using Centurion, K241R centrifuge (UK). Evaporation of samples was carried out by using a rotating concentrator that contains a vacuum pump (DVP-TYRO-12, Germany), trap for solvents (CHRIST-CT-02-50, Germany) and rotor (CHRIST-RVC 2-18 CD-plus, Germany).

### Preparation of the stock and working standard solutions

For preparation of stock standard solution, 25.0 mg of vilanterol standard was accurately weighed and placed in a 25.0 mL volumetric flask, dissolved and completed to volume with acetonitrile, resulting in a final concentration of 1.0 mg mL^−1^. A working standard solution of concentration 80.0 µg mL^−1^ was prepared by accurately transferring 800.0 µL from the stock standard solution into a 10.0 mL volumetric flask, and the volume was completed to the mark using acetonitrile. The stock standard solution was kept at 4 °C.

### Chromatographic conditions

Chromatographic analysis was achieved using Hypersil gold C18 column, 2.2 μm (2.1 × 100 mm) utilizing gradient mobile phase composed of mixture of (A) acetonitrile and (B) deionized water, with the pH of the latter adjusted to (5.0) using orthophosphoric acid. The ratio of (A) and (B) was (30: 70%; *v*/*v*) at the beginning of the run and changed throughout the course of the run till reach (70: 30%; *v*/*v*) after 20 min. the flow rate was 0.50 mL min^−1^ and analyses were carried out at ambient temperature with detection at 210 nm. The volume of injection was 10.0 µL. A mass spectrometer equipped with a triple quadrupole detector, operating in electrospray ionization (ESI) mode, was employed. The sample was directly injected into the detector, while maintaining a source temperature of 130 °C, a desolvation temperature of 400 °C, and a desolvation gas flow rate of 550 L/hour. The capillary voltage was set at 3 kV, and the cone voltage was maintained at 20 V.

### Calibration curve of vilanterol

Aliquots 1.0, 10.0, 50.0, 100.0, 200.0, 400.0, 600.0, 800.0 and 1000.0 µL were accurately transferred from the stock standard solution into a series of 10.0 mL volumetric flasks. The volume was completed to the mark using acetonitrile. A 10.0 µL volume of each solution was injected in triplicate in UPLC under the chromatographic conditions previously mentioned. The calibration curve was developed by plotting the average integrated peak area against the corresponding concentrations of vilanterol. The regression equation and coefficient (R^2^) were determined.

### Validation of the proposed method

The method was validated based on the International Conference on Harmonization (ICH)^30^ guidelines in terms of linearity, limit of detection (LOD), limit of quantification (LOQ), specificity, precision, accuracy, robustness and system suitability.

The method linearity was determined by injecting the nine concentrations used in the preparation of the calibration curve each for three times, and the results were assessed through the R^2^ of the calibration curve. LOD, which describes the minimum concentration that can be detected, and LOQ which describes the minimum concentration that can be quantified; were calculated by Eqs. (1) and (2), respectively:

1 $$LOD = 3 \times S/N$$

2 $$LOQ = 10 \times S/N$$

where S/N is the signal to noise ratio.

Specificity was determined by the ability of the method to determine the drug with a sharp and uniform peak, without any interference at the retention time of the drug.

Intra-day and inter-day and precision were evaluated by injecting concentrations of 1.0, 40.0 and 80.0 µg mL^−1^ in triplicates in the same day and three different days, respectively. Then, percentage relative standard deviation (% RSD) was calculated.

Accuracy of the method was evaluated by calculating the percentage recovery (% R) of concentrations of 1.0, 40.0, and 80.0 µg mL^−1^ injected in triplicates using Eq. (3):

3 $$\% R = Found\;concentration/Nominal\;concentration \times 100$$

Robustness determines the ability of the method to be not affected by deliberate changes in the chromatographic conditions. The results were evaluated by the % RSD.

System suitability parameters of the proposed method were tested, such as tailing factor, capacity factor, theoretical plates count and the height equivalent to theoretical plate.

### Forced degradation study and degradation kinetics.

Forced degradation study was done in accordance with the ICH guidelines in terms of acid, base, oxidation, thermal, and photolytic conditions^31^. The degradation products were subjected to structural elucidation using mass spectrometry to determine the pathway of the degradation and identify the degradation products. Degradation kinetics was implemented for the conditions that showed drug degradation, and the degradation parameters; degradation rate constant (K), half-life time (t_1/2_) and shelf-life time (t_90_); were calculated.

#### Acid and base-induced degradation

In 10.0 mL volumetric flasks each containing 5.0 mL of vilanterol solution of a concentration of 160 µg mL^−1^, a volumes of 0.25, 1.25 and 2.50 mL of 4.0 M HCl were transferred separately. The mixtures were reserved at room temperature for 2, 4, 8 and 24 h. After each time interval, samples were neutralized using an appropriate volume of 4.0 M NaOH, and then the volume was completed to the mark using acetonitrile so, the final concentration of vilanterol was 80.0 µg mL^−1^ in 0.10, 0.50 and 1.0 M HCl separately. These experiments were repeated at a higher temperature of 70.0 °C for 5, 15, 30, 60 and 120 min to determine the drug degradation kinetics while keeping the concentration of the drug, NaOH and HCl constant.

Alkaline degradation was performed in a similar way using 0.25, 1.25 and 2.50 mL of 4.0 M NaOH and neutralization was carried out using an appropriate volume of 4.0 M HCl. The forced degradation studies under acidic and basic conditions were performed in dark conditions in order to exclude the effect of light. 10.0 µL of the neutralized solutions; for both acidic and basic degradation; were injected into the column, and the chromatograms were run under the optimum conditions.

#### Hydrogen peroxide-induced degradation

In 10.0 mL volumetric flasks, 5.0 mL of 6.0 and 30.0% (*v*/*v*) H_2_O_2_ were added separately to a 5.0 mL of vilanterol solution at a concentration of 160.0 µg mL^−1^ to reach final concentrations of vilanterol 80.0 µg mL^−1^ in 3.0 and 15.0% (*v*/*v*) H_2_O_2_, respectively. The mixtures were kept at room temperature for 2, 4, 8 and 24 h. This experiment was repeated at higher temperature of 70.0 °C for 5, 15, 30, 60, 120, and 240 min to determine the drug degradation kinetics while keeping the concentration of the drug and H_2_O_2_ constant. 10.0 µL of the solutions were injected into the column, and the chromatograms were run under the optimum conditions.

#### Thermal and photolytic degradation

An accurately weighed 10.0 mg of the dry powdered drug was placed in an oven at 60.0 °C for a duration of 72 h to study the effect of heat on the degradation of vilanterol. The powder was dissolved in 10.0 mL acetonitrile then a volume of 800.0 µL was transferred into 10.0 mL volumetric flask and diluted with acetonitrile to prepare a solution of concentration of 80.0 µg mL^−1^.

To study the photochemical stability, an amount of 10.0 mg of the dry powder of the drug was exposed to UV for 72 h. The powder was treated as mentioned in the thermal degradation to prepare a solution of concentration of 80.0 µg mL^−1^. Ten microliters of the resultant solutions were injected to the column and the chromatograms were run under optimum conditions.

### Assay of vilanterol and degradation product in human urine sample

For sample preparation in 10.0 mL volumetric flask, aliquots of 1.0 mL of urine samples were collected from fasting individuals who are in good health and without any pre-existing medical conditions were spiked with 10.0, 50.0, 100.0, 200.0, 400.0, 600.0, 800.0 and 1000.0 µL of 1.0 mg mL^−1^ vilanterol stock solution separately. Each concentration was spiked with 1.0 mL of oxidative degradation product obtained by vilanterol degradation using 15.0% H_2_O_2_ for 4 h at 70.0 °C. The spiked urine samples were completed to the mark using de-ionized water adjusted to pH 5.00 by orthophosphoric acid. So, the final prepared drug concentrations were 1.0, 5.0, 10.0, 20.0, 40.0, 60.0, 80.0 and 100.0 µg mL^−1^. A similar procedure was carried out by spiking aliquots of 1.0 mL of urine samples with the same concentrations of the drug and 1.0 mL of acidic and basic degradation products obtained by vilanterol degradation using 1.0 M HCl and 1.0 M NaOH for 2 h at 70.0 °C separately. All samples were left for 30 min, vortexed for 5 min and filtered using 0.45 µm nylon syringe filter. Subsequently, 10 µL of the filtered solutions were injected to UPLC.

Intra and the inter-day precision were evaluated by injecting concentrations of 1.0, 20.0 and 60.0 µg mL^−1^ in triplicates in three different days and in the same day, respectively. Then, % RSD was calculated. Accuracy of the method was evaluated by calculating % R of concentrations of 1.0, 20.0 and 60.0 µg mL^−1^ injected in triplicates.

## Results and discussion

### Method development

During method optimization, several chromatographic conditions were attempted using a Hypersil gold C18 column 2.2 μm particle size (2.1 i.d. × 100 mm). The mobile phase consisted of methanol and water in different ratios, where (50: 50; *v*/*v*), (75: 25; *v*/*v*) and (90: 10; *v*/*v*) were tested using isocratic mode. The results indicated that increasing the % of methanol improved peak uniformity and intensity. As a result, methanol was used with de-ionized water with ratio (90: 10; *v*/*v*). The pKa of vilanterol was 9.90 and 8.90 for the acidic and basic part, respectively^32^. Accordingly, de-ionized water with pH adjusted to (3.0) and (5.0) using orthophosphoric acid were tried and there was no difference in the peak symmetry and intensity. So, de-ionized water with pH 5.0 was selected. Acetonitrile was tried instead of methanol with the same ratio, and this resulted in decreasing the retention time of the drug and decreasing the pump pressure due to the higher elution power and lower column pressure of acetonitrile than methanol^33^ so, acetonitrile was selected as an organic solvent with de-ionized water adjusted to pH 5.0 using orthophosphoric acid with ratio (90:10; *v*/*v*). Attempts were made to separate vilanterol from its degradation products using the aforementioned method. However, it was observed that the resolution of the separation was poor, and the peaks of the analyte and its degradation products were found to overlap, indicating a lack of selectivity in the separation process. So, gradient elution was adopted, using a mobile phase consisting of acetonitrile (A) and de-ionized water adjusted to pH 5.0 using orthophosphoric acid (B). The run started with the mobile phase (A):(B) with a ratio of (30: 70%; *v*/*v*), then, percentage of (A) gradually increased till it reached (70:30%; *v*/*v*). This method was able to successfully separate vilanterol from its degradation products with good resolution and sharp and uniform peaks. The gradient time was 20 min at the beginning and under these conditions degradation products were eluted early and vilanterol eluted at 17.4 min. Based on this information, it was determined that the degradation products appeared with a high percentage of (B) in the mobile phase, while the presence of a high percentage of (A) favored the elution of vilanterol. In addition gradient elution using (A):(B) in a ratio of ratio of (70.0:30.0; *v*/*v*) at the beginning of the run and changing the ratio till reach ratio of (30.0:70.0; *v*/*v*) led to overlapping between the peak of vilanterol and its degradation products. Also, a stepwise gradient elution method was employed. Initially, a mobile phase ratio (A):(B), (30.0:70.0; *v*/*v*) was utilized for the first 5 min to elute the degradation products, followed by a transition to a ratio of (70.0:30.0; *v*/*v*) over the subsequent 10 min to elute vilanterol. Successful elution of vilanterol was achieved within 12 min. Subsequently, the mobile phase was adjusted back to a ratio of (30.0:70.0; *v*/*v*) for the final 5 min to condition the column for subsequent runs. As a result, the total run time was also 20 min. All the trials were carried out at 210 nm to obtain the highest peak intensity, as this is the λ_max_ of vilanterol observed from the photo diode array detector (PDA). The column temperature was adjusted at 25 °C and increasing the temperature to 30 °C was tried and it did not have a great impact on the separation or the run time so, the selected temperature was 25 °C. After the evaluation of various chromatographic conditions and trials, it was decided that the optimum chromatographic method for the separation of vilanterol from its degradation products was a gradient mobile phase composed of a mixture of (A) acetonitrile and (B) deionized water, with the pH of the latter adjusted to 5.0) using orthophosphoric acid. The ratio of (A) and (B) was (30:70%; *v*/*v*) at the beginning of the run and changed throughout the course of the run till reach (70:30%; *v*/*v*) at the end of the run with a flow rate of 0.5 mL min^−1^ and the column oven was adjusted at 25 °C with samples injection volume of 10.0 µL and the detection was at 210 nm.

### Method validation

#### Linearity, limit of detection and limit of quantification

The calibration curve of vilanterol was linear in range from 0.10 to 100.0 µg mL^−1^ as represented in Fig. S1. The regression equation, R^2^, LOD and LOQ were described in Table S1.

#### Accuracy and precision

The proposed method showed high accuracy as the % R was in the range of 99.45–100.02% and good precision as the % RSD was less than 2.0%, as shown in Table S2.

#### Specificity

The method was able to determine the analyte with a sharp and uniform peak at a retention time of 17.4 min without the presence of any interfering substance, as shown in Fig. S2a.

#### Robustness

The findings indicated that the method is robust and not significantly influenced by minor variations in the factors mentioned earlier, which is apparent from the low % RSD values presented in Table S3.

#### System suitability

The system suitability parameters were calculated as tailing factor, capacity factor, theoretical plates count, and height equivalent to each theoretical plate. The outcomes of these tests for the proposed method are presented in Table S4.

### Degradation behavior

#### Acid-and base-induced degradation

Subjecting vilanterol standard material to 0.10, 0.50 and 1.0 M HCl and NaOH at ambient temperature for different time intervals which were 2, 4, 8 and 24 h did not show the presence of any degradation product as represented in Fig. S2b, c. This indicates the high stability of the drug under these conditions.

Subjecting vilanterol to heightened stress conditions at a temperature of 70.0 °C for a duration of 30 min using 0.10, 0.50, and 1.0 M HCl and NaOH resulted in the emergence of consequent degradation products, thus warranting an assessment of the drug degradation kinetics at time intervals of 5, 15, 30, 60, and 120 min for each concentration to determine its rate and percentage of decomposition. As presented in Table 1, the percentage of vilanterol degradation increased by increasing the concentration of NaOH and HCl at each time interval at 70.0 °C. This indicated that the drug is susceptible to more acidic and basic degradation by increasing the concentration of NaOH and HC. The chromatograms of vilanterol degradation using 0.10 M of NaOH at different time intervals at 70.0 °C showed a decrease in the peak area of vilanterol in addition to the appearance of two additional peaks DP_1_ and DP_2_ at 2.12 and 4.67 min, respectively. This indicated that vilanterol exhibits susceptibility to alkaline hydrolysis, resulting in degradation products that can be effectively resolved as shown in the chromatogram in Fig. S3. While for acidic stress conditions, the chromatograms of vilanterol degradation using 0.10 M of HCl and different time intervals at 70.0 °C showed a decrease in the peak area of vilanterol in addition to the appearance of two additional peaks DP_3_ and DP_4_ at 1.98 and 4.82 min, respectively. This indicated that vilanterol exhibits susceptibility to acidic hydrolysis as represented in Fig. S4. The resultants DP_1_, DP_2_, DP_3_ and DP_4_ were characterized through mass spectrometry.

**Table 1:** Vilanterol degradation percentage under different degradation conditions at 70 °C.

Degradation condition		Percentage of vilanterol degradation
Acid		
0.10 M	5 min	20.35
	15 min	37.46
	30 min	56.25
	60 min	75.35
	120 min	93.16
0.50 M	5 min	22.53
	15 min	39.26
	30 min	59.94
	60 min	78.41
	120 min	95.91
1.0 M	5 min	25.62
	15 min	44.16
	30 min	65.62
	60 min	82.45
	120 min	100.00
Base		
0.10 M	5 min	25.61
	15 min	36.32
	30 min	64.34
	60 min	88.25
	120 min	97.06
0.50 M	5 min	29.21
	15 min	40.23
	30 min	69.14
	60 min	94.02
	120 min	100.00
1.0 M	5 min	35.31
	15 min	49.13
	30 min	75.37
	60 min	96.32
	120 min	100.00
15.0% H_2_O_2_	5 min	6.25
	15 min	15.15
	30 min	35.44
	60 min	56.25
	120 min	80.32
	240 min	100.00

#### Hydrogen peroxide-induced degradation

Exposing vilanterol to 3.0 and 15.0% of H_2_O_2_ at ambient temperature for 2, 4, 8 and 24 h did not reveal the presence of any degradation product as represented in Fig. S2d. Subsequent to this, the temperature was raised to 70.0 °C for duration of 30 min under the same H_2_O_2_ concentrations. The obtained results demonstrated no discernible impact on the drug when using H_2_O_2_ concentration of 3.0% whereas an additional peak (DP_5_) was observed upon using 15.0% of H_2_O_2._ Accordingly, degradation kinetics was carried out at discrete time intervals of 5, 15, 30, 60, 120 and 240 min using 15.0% of H_2_O_2_. Analysis of the results revealed that the peak of DP_5_ eluted after 1.48 min increased with an increase in time while the drug peak decreased as represented in Fig. S5. The resultant DP_5_ was characterized using mass spectrometry. Also, the percentage of vilanterol degradation at different time intervals using 15.0% H_2_O_2_ was summarized in Table 1.

#### Thermal and photolytic degradation

The chromatograms of thermal and photolytic stress conditions did not reveal any change in the peak area of vilanterol and did not show the presence of any additional peaks. This revealed the stability of vilanterol to both thermal and photolytic stress conditions as represented in Fig. S2e, f.

#### Determination of kinetics parameters

**Figure 2:**
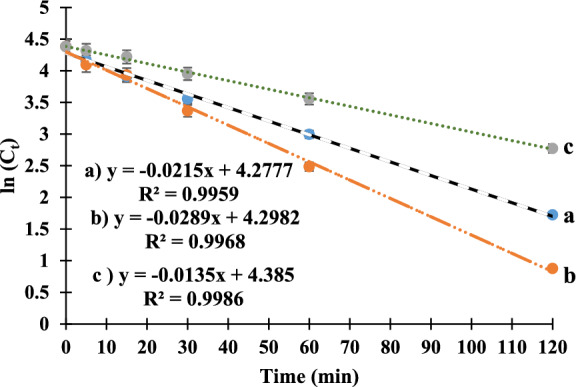
First-order degradation graph for 0.10 M acidic degradation (**a**), 0.10 M basic degradation (**b**) and oxidative degradation using 15.0% H_2_O_2_ all at 70.0 °C (**c**).

The degradation kinetics parameters were assessed for acidic, basic, and oxidative degradation processes. (K), (t_1/2_) and (t_90_) were calculated after vilanterol exposure to acidic, basic and oxidative stress conditions using Eqs. (4–6) where, C_t_ is the remaining drug concentration after a certain time which has been calculated from the peak area of the remaining drug at certain time µg mL^−1^ and °C is the initial drug concentration µg mL^−1^. The results of the kinetics parameters were represented in Table 2. Upon plotting ln(C_t_) versus time (t) for acidic, basic and oxidative stress conditions, a linear relationship was obtained as represented in Fig. 2. Based on this information, it is assumed that the degradation behavior adheres to first-order degradation kinetics. This means that the reaction rate for the degradation of the drug under various conditions can be established by observing the decrease in the initial drug concentration over time. In other words, the rate of degradation is directly proportional to the decrease in the initial concentration of the drug as a function of time. Within the studied period, the calculated degradation parameters for basic degradation pathway showed higher values than the acidic one and the oxidative degradation pathway showed the least values which indicated that the degradation of the drug was fastest under basic conditions and slowest under oxidative conditions.

4 $$ln \left( {C_{t} } \right) = ln \left( {C^\circ } \right) - Kt$$

5 $$t_{1/2} = 0.693/K$$

6 $$t_{90} = 0.105/K$$

**Table 2:** Kinetics parameters of vilanterol acidic, basic and oxidative degradation at 70.0 °C.

Kinetics parameter	Acidic degradation (0.10 M)	Basic degradation (0.10 M)	Oxidative degradation (15.0%)
K (min^−1^)	0.0215	0.0289	0.0135
t_1/2_ (min)	32.23	23.98	51.33
t_90_ (min)	4.88	3.63	7.78

### Structural elucidation of the degradation products

**Figure 3:**
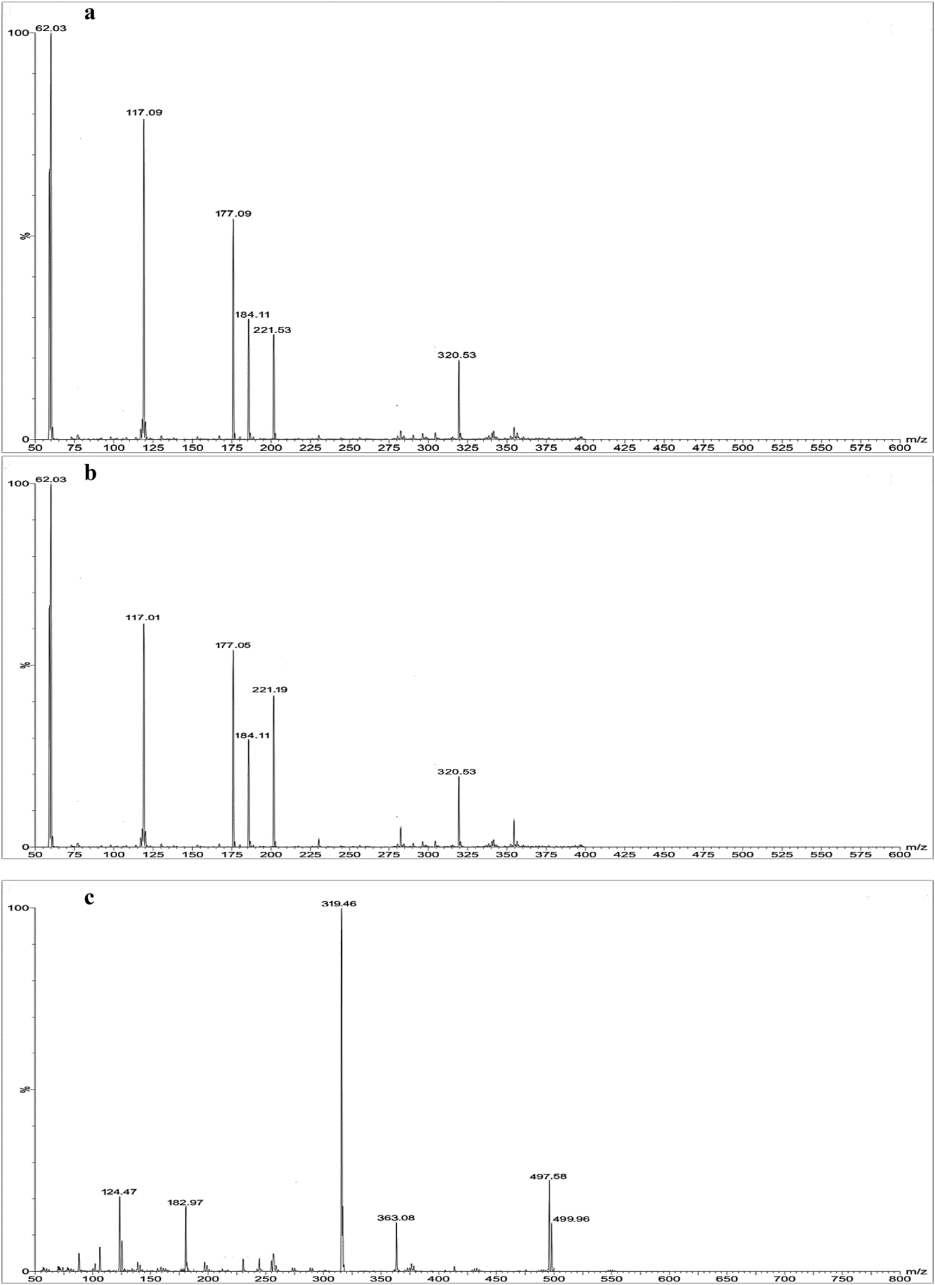
Mass scan spectra of [M + H] ^+^ of the acidic (**a**), basic (**b**) and oxidative (**c**) degradation products of vilanterol.

**Figure 4:**
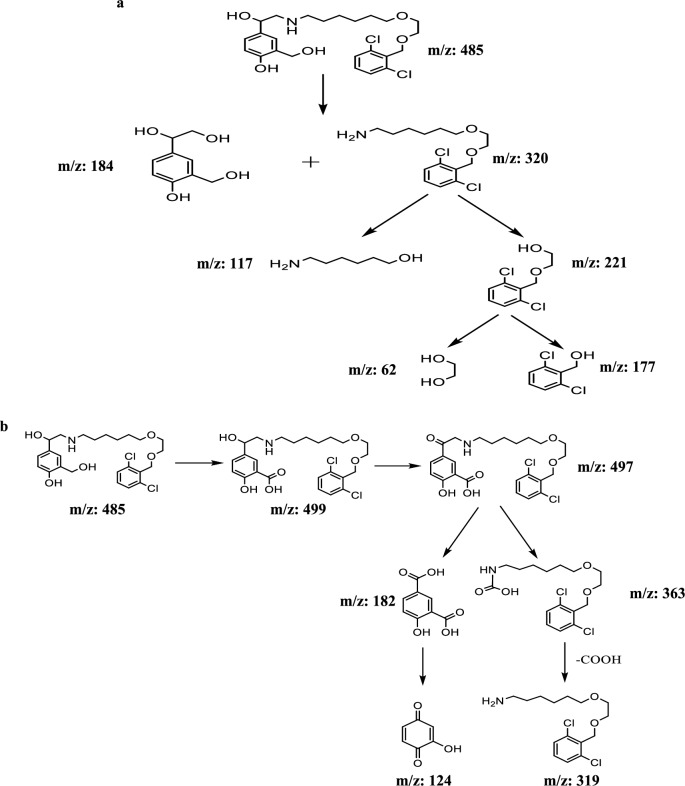
The suggested acidic and basic degradation pathway (**a**) and oxidative degradation pathway (**b**) for vilanterol.

Through the application of mass spectrometry scanning to the various degradation samples; subjected to acidic, basic, and oxidative stress conditions; the identification of degradation products m/z values were attained, as depicted in Fig. 3. The structure analysis was conducted in ESI–MS positive ion mode. The mass spectrum of vilanterol intact drug indicated a substantial relative abundance vilanterol peak [M + H] ^+^ ion with m/z value of 486 and 488 due to the presence of chlorine which has m/z value of 35 or 37. Figure 4a was devised in order to illustrate the pathway suggested for vilanterol degradation and the structure elucidation of the degradants under acidic and basic hydrolysis stress conditions which showed the same degradation behavior. The degradation involved the detachment of 1-(4-hydroxy-3-(hydroxymethyl)phenyl)ethane-1,2-diol with m/z value of 184 and 6-(2-((2,6-dichlorobenzyl)oxy)ethoxy)hexan-1-amine with m/z value of 320. The later one was further degraded to form 6-aminohexan-1-ol with m/z value of 117 and 2-((2,6-dichlorobenzyl)oxy)ethan-1-ol with m/z value of 221 which degraded to produce ethane-1,2-diol with m/z value of 62 and (2,6-dichlorophenyl)methanol with m/z value of 177. The suggested acidic and basic stress degradation protocol that was implemented demonstrated the capacity to generate 6-(2-((2,6-dichlorobenzyl)oxy)ethoxy)hexan-1-amine with m/z value of 320 that resemble that formed by endogenous biotransformation of vilanterol by human body via N-dealkylation followed by glucuronidation, and were primarily excreted in urine^34^.

Figure 4b was devised in order to illustrate the pathway suggested for vilanterol degradation and the structure elucidation of the degradants under oxidative stress conditions, which involved the detachment of 5-(2-((6-(2-((2,6-dichlorobenzyl)oxy)ethoxy)hexyl)amino)-1-hydroxyethyl)-2-hydroxybenzoic acid with m/z value of 499 which further oxidized to produce 5-((6-(2-((2,6-dichlorobenzyl)oxy)ethoxy)hexyl)glycyl)-2-hydroxybenzoic acid with m/z value of 497 then, the later was oxidized to form two fragments which were 4-hydroxyisophthalic acid with m/z value of 182 and (6-(2-((2,6-dichlorobenzyl)oxy)ethoxy)hexyl)carbamic acid with m/z value of 363. The first one was oxidized to produce 2-hydroxycyclohexa-2,5-diene-1,4-dione with m/z value of 124 and the second one was oxidized to produce 6-(2-((2,6-dichlorobenzyl)oxy)ethoxy)hexan-1-amine with m/z value of 319. The suggested oxidative stress degradation protocol that was implemented demonstrated the capacity to generate 5-(2-((6-(2-((2,6-dichlorobenzyl)oxy)ethoxy)hexyl)amino)-1-hydroxyethyl)-2-hydroxybenzoic acid with m/z value of 499 that resemble that formed by endogenous biotransformation of vilanterol by human body via oxidation followed by elimination in urine^34^.

### Assay of vilanterol and degradation products in human urine sample

**Figure 5:**
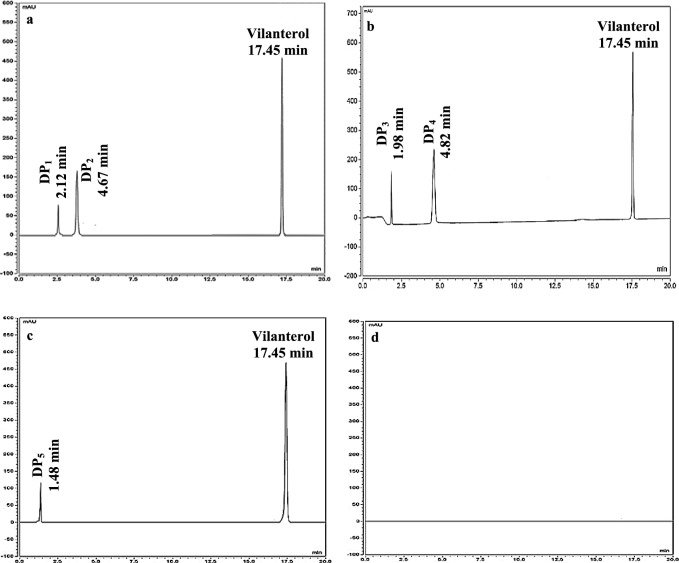
UPLC chromatograms of 80.0 µg mL^−1^ vilanterol standard in the presence of metabolite produced by basic degradation (**a**), acidic degradation (**b**), oxidative degradation (**c**), spiked to human urine matrix and blank urine (**d**).

In light of WADA’s ban on vilanterol among athletes^16^, the present study aimed to develop a method for the quantitative analysis of vilanterol in the presence of its endogenously produced metabolites in urine. The findings indicated that the proposed method exhibits satisfactory linearity for the quantification of vilanterol in the presence of its in house produced metabolites, as depicted in Fig. S6, and demonstrated a high degree of accuracy and precision, as outlined in Table 3. The proposed method showed a good resolution in the separation of vilanterol from the metabolites produced by acidic, basic, and oxidative degradation as represented in the chromatograms of Fig. 5 in addition to the ability of the analytical method to purify urine samples from any impurities as presented in the blank urine chromatogram Fig. 5. The outcomes of this study align with the collective endeavors of various sports organizations to mitigate the incidence and prevalence of doping in sports. The developed protocol enabled the generation of vilanterol metabolites that resemble those formed by endogenous biotransformation, as well as the accurate quantification of vilanterol in the presence of its metabolites in human urine. These findings have significant implications for the detection of vilanterol doping through the identification of the drug and/or its metabolite in human urine.

**Table 3:** Accuracy, intra-day and inter-day precision of vilanterol standard in the presence of its metabolites in human urine matrix using the proposed UPLC method.

Concentration (µg mL^-1^)	% R*			Intra-day precision* % RSD			Inter-day precision* % RSD		
	Acid	Base	Oxidation	Acid	Base	Oxidation	Acid	Base	Oxidation
1.00	98.35	98.62	98.47	0.510	0.341	0.490	0.683	0.441	0.381
20.00	99.60	98.99	98.92	0.622	0.415	0.521	0.922	0.520	0.684
60.00	98.97	99.01	99.21	0.241	0.363	0.382	0.511	0.465	0.470

*Average of 3 determinations.

Finally, a comparative analysis between our newly developed stability indicating assay method and other stability studies previously reported in the literature^24–26^ was developed and summarized in Table S5. Where none of the stability indicating methods reported for vilanterol includes structural elucidation for the degradation products using MS. Moreover, none of the reported methods include the production of metabolites under laboratory degradation conditions.

## Conclusion

A sensitive, accurate and precise stability indicating UPLC method was developed and validated for the analysis of vilanterol in the presence of its degradation products. Acidic, basic, and oxidative stress conditions showed the presence of degradation products while the drug was not affected by thermal and photolytic stress conditions. Kinetics parameters which were (K), half-life time (t_1/2_) and shelf-life time (t_90_) were calculated, and it was observed that the degradation rate followed first order reaction. Structural elucidation of acidic, basic, and oxidative degradants was performed using mass spectrometer. The results showed the presence of degradants similar to vilanterol metabolites formed by human body. The proposed method was used for the quantification of vilanterol in the presence of its degradation products in human urine with high accuracy and precision. This method can be used for the revealing of vilanterol doping either by the detection of its intact form or its metabolites or both.

### Supplementary Information

Supplementary Information.

## Data Availability

Data will be made available on request. The request for materials should be addressed to M.T. (mohamed.tarek@bue.edu.eg).
