# Supplementary material for: Stability-indicating UPLC assay coupled with mass spectrometry for the analysis of vilanterol degradation products in human urine
Source: Sci Rep. 2024 Jan 30;14:2439. doi: 10.1038/s41598-024-52664-6 (PMC10824719; doi:10.1038/s41598-024-52664-6)
Supplement: Supplementary file 1 — Supplementary Information. [file 41598_2024_52664_MOESM1_ESM.docx]

**Stability-indicating UPLC assay coupled with mass spectrometry for the analysis of vilanterol degradation products in human urine.**

Mohamed Tarek^1, 2^, Hebatallah A. Wagdy^1, 2**^, Maha A. Hegazy^3*^, Nermine S. Ghoniem^3^

^1^Pharmaceutical Chemistry Department, Faculty of Pharmacy, The British University in Egypt (BUE), Cairo, Egypt.

^2^Health Research Center of Excellence; Drug Research and Development Group, Faculty of Pharmacy, The British University in Egypt, Cairo, Egypt.

^3^Analytical Chemistry Department, Faculty of Pharmacy, Cairo University, Kasr-El Aini Street, 11562 Cairo, Egypt.

*** Corresponding Author**

Maha A. Hegazy

Department of Analytical Chemistry, Faculty of Pharmacy, Cairo University.

E-mail address: [Maha.hegazy@pharma.cu.edu.eg](mailto:Nermine.ghoniem@pharma.cu.edu.eg)

**** Co-corresponding Author**

Hebatallah A. Wagdy

Department of Pharmaceutical Chemistry, Faculty of Pharmacy, The British University in Egypt.

E-mail address: [Hebatallah.wagdy@bue.edu.eg](mailto:Hebatallah.wagdy@bue.edu.eg)

**List of tables:**

**Table S1:** Linearity, LOD and LOQ of vilanterol standard using the proposed UPLC method.

**Table S2:** Accuracy, intra-day and inter-day precision of vilanterol standard using the proposed UPLC method.

**Table S3:** Robustness of the proposed UPLC method to different investigated factors.

**Table S4:**  System suitability parameters of the proposed UPLC method.

**Table S5**: A comparison between the proposed method and the previously reported stability studies in literature.

| **Table S1:** Linearity, LOD and LOQ of vilanterol standard using the proposed UPLC method | | | | |
| --- | --- | --- | --- | --- |
| **Drugs** | **Regression equation *** | **R^2^** | **LOD (µg mL^-1^)** | **LOQ (µg mL^-1^)** |
| **Vilanterol** | $Y=0.2664x+0.1275$ | 0.9991 | 0.03 | 0.10 |
| * Regression equation was calculated from average peak area | | | | |

| **Table S2:** Accuracy, intra-day and inter-day precision of vilanterol standard using the proposed UPLC method | | | | |
| --- | --- | --- | --- | --- |
| **Drug** | **Concentration**  **(µg mL^-1^)** | **% R*** | **Intra-day precision ***  **% RSD** | **Inter-day precision ***  **% RSD** |
| **Vilanterol** | 1.00 | 99.45 | 0.041 | 0.391 |
|  | 40.00 | 99.60 | 0.243 | 0.482 |
|  | 80.00 | 100.02 | 0.345 | 0.694 |
| *Average of 3 determinations | | | | |

| **Table S3:** Robustness of the proposed UPLC method to different investigated factors | |
| --- | --- |
| **Effect of temperature change** | |
| Temperature change (^◦^C) | % RSD * |
| 25.0±1.0 | 0.651-0.890 |
| **Effect of % Aqueous: Organic phases change** | |
| % Organic: Aqueous | % RSD * |
| (90: 10) ±1.0 | 0.582-0.923 |
| **Effect of pH change** | |
| pH | % RSD * |
| 5.0±0.1 | 0.631-0.850 |
| **Effect of wavelength change** | |
| Wavelength (nm) | % RSD * |
| 210±2 | 0.182-0.230 |
| **Effect of flow rate change** | |
| Flow rate (mL min^-1^) | % RSD * |
| 0.50±0.10 | 0.312-0.440 |
| *Average of 3 times | |

| **Table S4:**  System suitability parameters of the proposed UPLC method | | |
| --- | --- | --- |
| **Parameter** | **Vilanterol** | **Reference value [1]** |
| **Retention time (t_R_) (min)** | 17.45 | ------ |
| **Tailing factor (T_f_)** | 1.05 | Less than 2 |
| **Capacity Factor (K’)** | 3.45 | >2 |
| **Number of theoretical plates (N)** | 3150 | >2000 |
| **Height equivalent to a theoretical plate (HETP)** | 0.0003 | The smaller the value the higher the column efficiency |

| **Table S5**: A comparison between the proposed method and the previously reported stability studies in literature | | | | | | | | | | |
| --- | --- | --- | --- | --- | --- | --- | --- | --- | --- | --- |
|  | | | | **Our proposed method** | | | **[2]** | | **[3]** | **[4]** |
| **Drugs** | | | | **Vilanterol** | | | **Vilanterol and umeclidinium** | | **Vilanterol and umeclidinium** | **Vilanterol and umeclidinium** |
| **Instruments** | | | | UPLC using PDA detector and mass spectrometer. | | | HPLC | | HPLC | HPLC |
| **Linearity**  **(µg mL^-1^)** | | | | 0.10 -100.00 | | | 15.625 - 93.75 | | 6.25 - 37.50 | 6.25 - 37.50 |
| **Stress conditions** | Acidic | | | 0.1, 0.5 and 1 M HCl at room temperature for 2, 8, 4 and 24 h and at 70.0^º^C for 5, 15, 30, 60 and 120 min. | | | 2 M HCl at 60^º^C for 30 min. | | 2 M HCl at 60^º^C for 30 min. | 2 M HCl at 60^º^C for 30 min. |
|  | Basic | | | 0.1, 0.5 and 1 M NaOH at room temperature for 2, 8, 4 and 24 h and at 70.0^º^C for 5, 15, 30, 60 and 120 min. | | | 2 M NaOH at 60^º^C for 30 min. | | 2 M NaOH at 60^º^C for 30 min. | 2 M NaOH at 60^º^C for 30 min. |
|  | Oxidative | | | 3 and 15 % H_2_O_2_ at room temperature for 2, 8, 4 and 24 h and at 70.0^º^C for 5, 15, 30, 60, 120 and 240 min. | | | 20 % H_2_O_2_ at 60^º^C for 30 min. | | 20 % H_2_O_2_ at 60^º^C for 30 min. | 20 % H_2_O_2_ at 60^º^C for 30 min. |
|  | Thermal | | | Oven at 60.0^º^C for 72 h. | | | Oven at 70.0^º^C for 24 h. | | Oven at 70.0^º^C for 24 h. | Oven at 70.0^º^C for 24 h. |
|  | Photolytic | | | UV lamp at 60.0^º^C for 72 h. | | | UV lamp at 70.0^º^C for 24 h. | | UV lamp at 70.0^º^C for 24 h. | UV lamp at 70.0^º^C for 24 h. |
| **Degradation kinetics** | Evaluated. | | | | | Not applicable. | | | Not applicable. | Not applicable. |
| **Kinetics parameters** | Evaluated | | | | | | Not applicable. | | Not applicable. | Not applicable. |
| **Structure elucidation of the degradants** | | Using mass spectrometer. | | | | | Not applicable. | | Not applicable. | Not applicable. |
| **Matrices of analysis** | | | Human urine. | | Dosage form. | | | Dosage form. | | Dosage form. |

**List of figures:**

**Fig. S1:** Calibration curve of vilanterol standard. Error bars are average of three determinations.

**Fig. S2:** UPLC chromatograms of 80.0 µg mL^-1^ vilanterol standard (**a**), subjected to 1.0 M HCl at ambient temperature (**b**), subjected to 1.0 M NaOH at ambient temperature (**c**), under oxidation using 15.0 % H_2_O_2_ at ambient temperature (**d**), thermal degradation (**e**) and photolytic degradation (**f**) using the optimum chromatographic conditions.

**Fig. S3:** UPLC chromatograms showing the degradation kinetics of 80.0 µg mL^-1^ vilanterol standard subjected to 0.10 M NaOH at 70.0^°^C at 5 min (**a**), 15 min (**b**), 30 min (**c**), 60 min (**d**) and 120 min (**e**) using the optimum chromatographic conditions.

**Fig. S4:** UPLC chromatograms showing the degradation kinetics of 80.0 µg mL^-1^ vilanterol standard subjected to 0.10 M HCl at 70.0^°^C at 5 min (**a**), 15 min (**b**), 30 min (**c**), 60 min (**d**) and 120 min (**e**) using the optimum chromatographic conditions**.**

**Fig. S5:** UPLC chromatograms showing the degradation kinetics of 80.0 µg mL^-1^ vilanterol standard subjected to 15.0 % H_2_O_2_ at 70.0^º^C at 5 min (**a**), 15 min (**b**), 30 min (**c**), 60 min (**d**), 120 min (**e**) and 240 min (**f**) using the optimum chromatographic conditions.

**Fig. S6:** Calibration curve of vilanterol in the presence of its metabolite produced by acidic degradation (**a)**, basic degradation (**b**) and oxidative degradation (**c**) spiked to human urine matrix.

**Fig. S1**

**
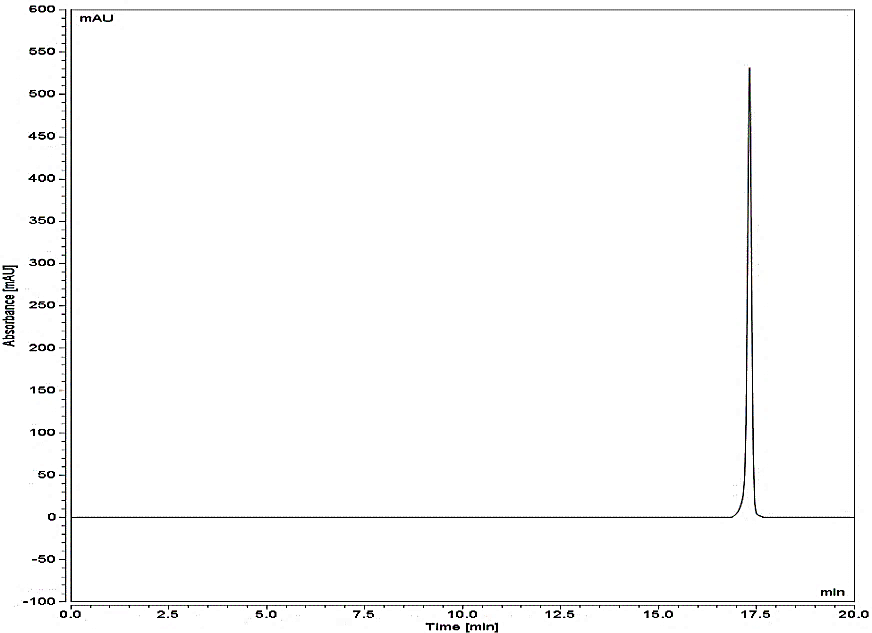

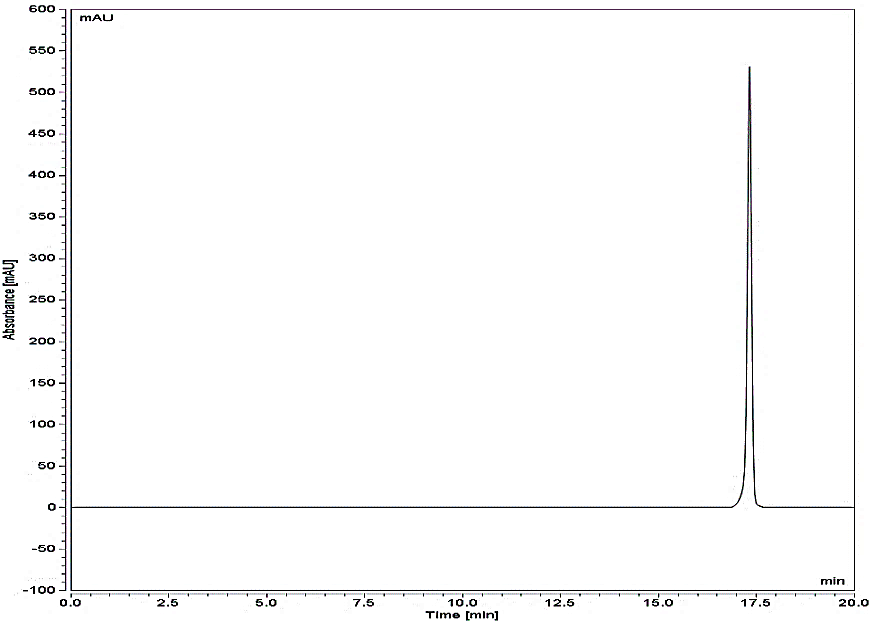
**

**Vilanterol**

**17.45 min**

**Vilanterol**

**17.45 min**

**b**

**a**

**
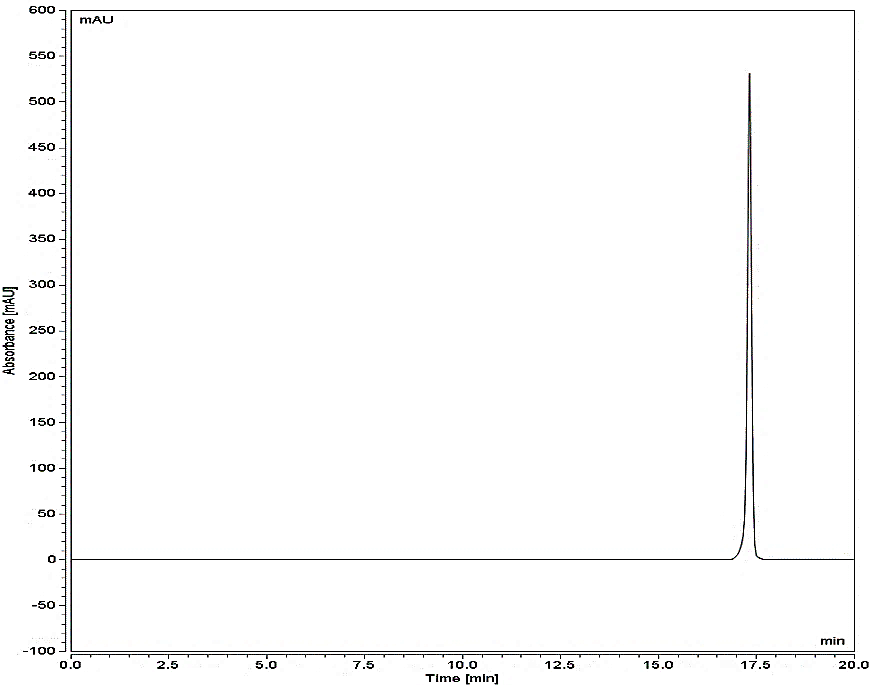

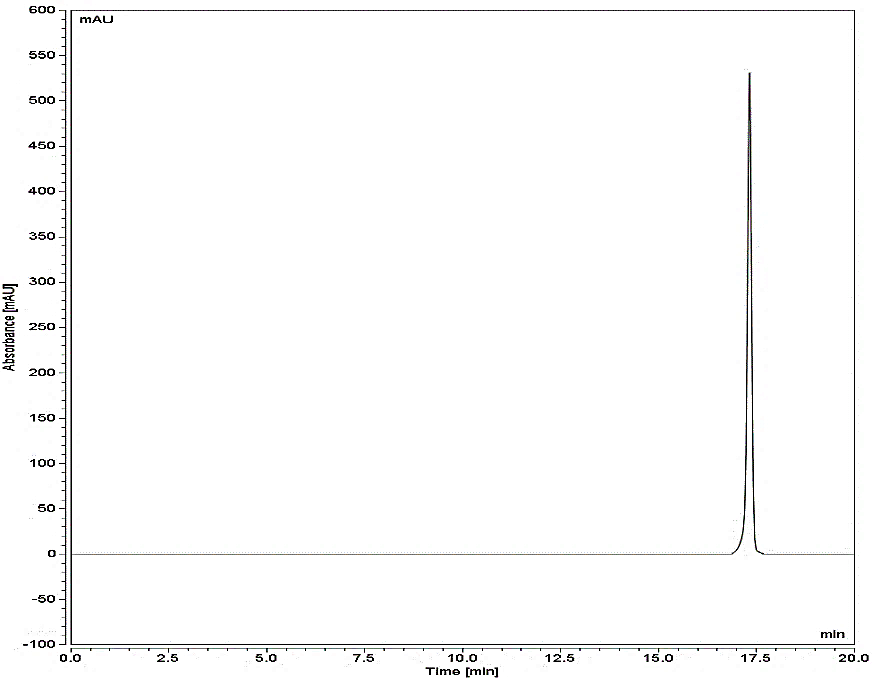
**

**d**

**c**

**Vilanterol**

**17.45 min**

**Vilanterol**

**17.45 min**

**
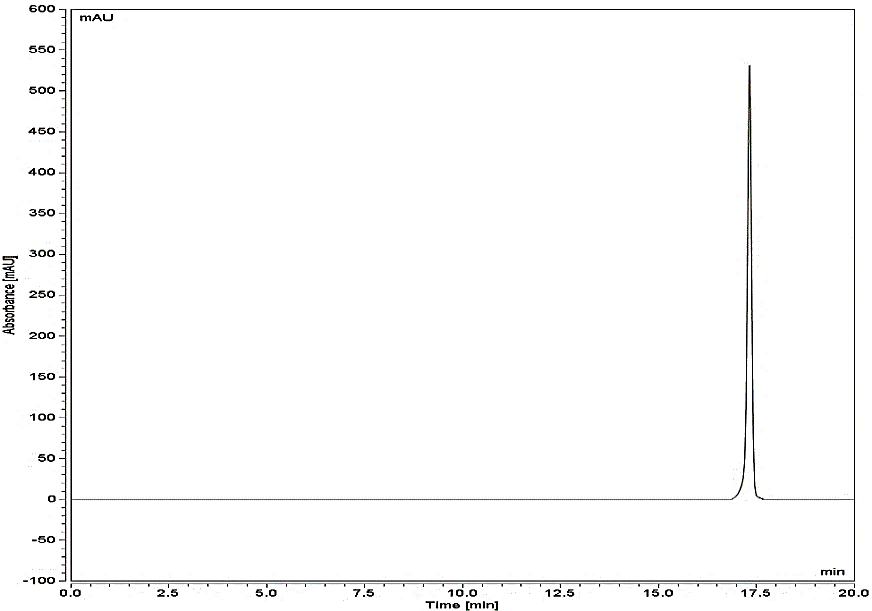
**

**
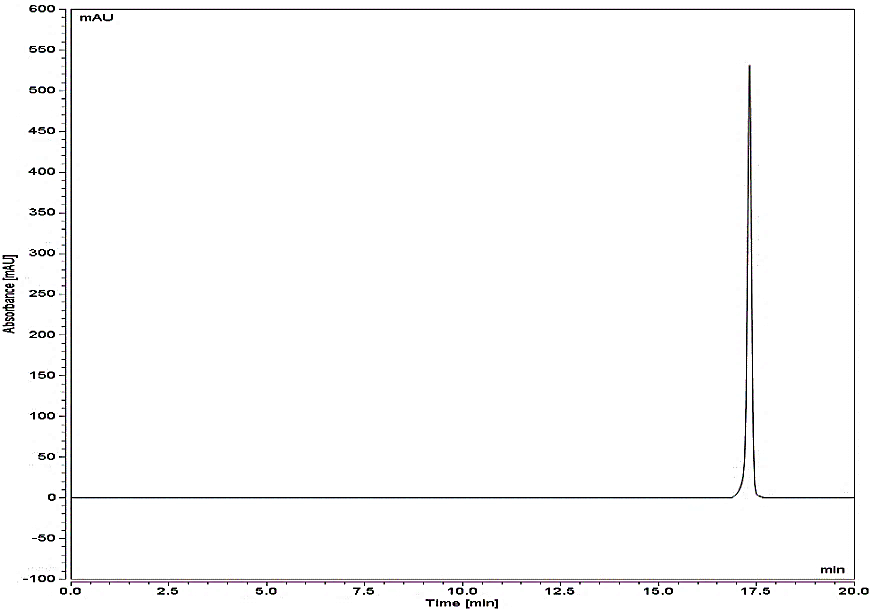
**

**f**

**e**

**Vilanterol**

**17.45 min**

**Vilanterol**

**17.45 min**

**Fig. S2**

**Vilanterol**

**17.45 min**

**Vilanterol**

**17.45 min**

**
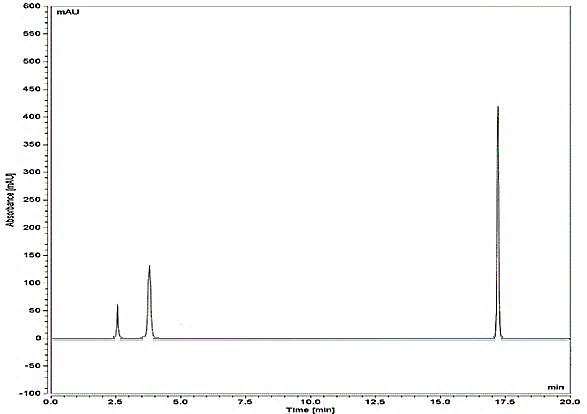

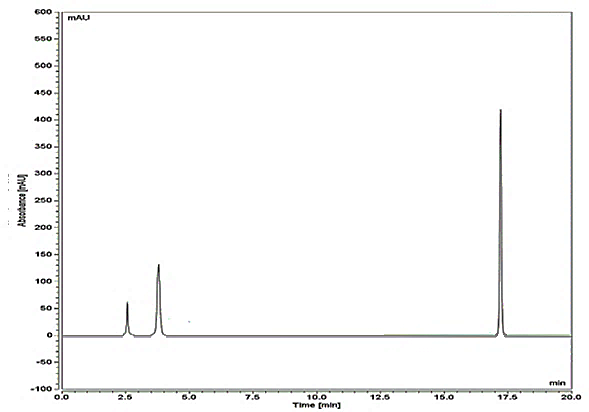
**

**DP_2_**

**4.67 min**

**DP_2_**

**4.67 min**

**DP_1_**

**2.12 min**

**DP_1_**

**2.12 min**

**b**

**a**

**
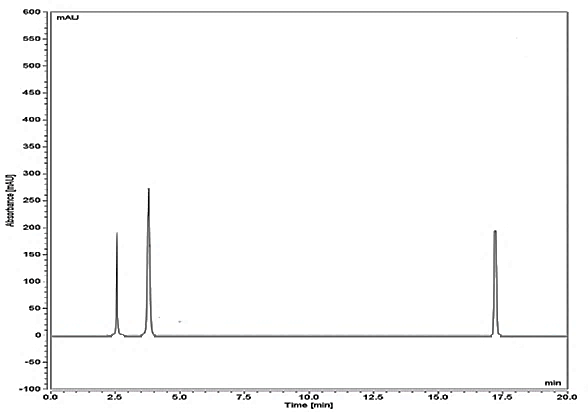
**

**
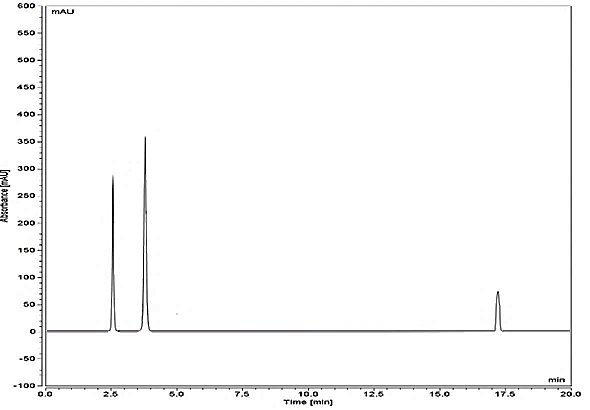
**

**DP_2_**

**4.67 min**

**c**

**DP_1_**

**2.12 min**

**d**

**DP_2_**

**4.67 min**

**DP_1_**

**2.12 min**

**Vilanterol**

**17.45 min**

**Vilanterol**

**17.45 min**

**
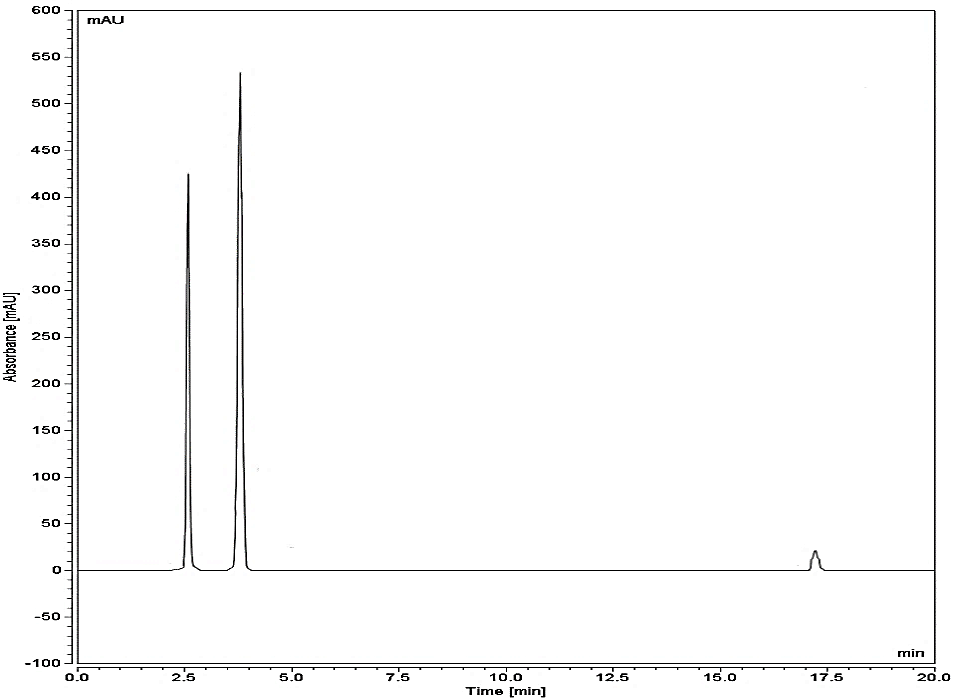
**

**DP_2_**

**4.67 min**

**DP_1_**

**2.12 min**

**e**

**Vilanterol**

**17.45 min**

**Fig. S3**

**
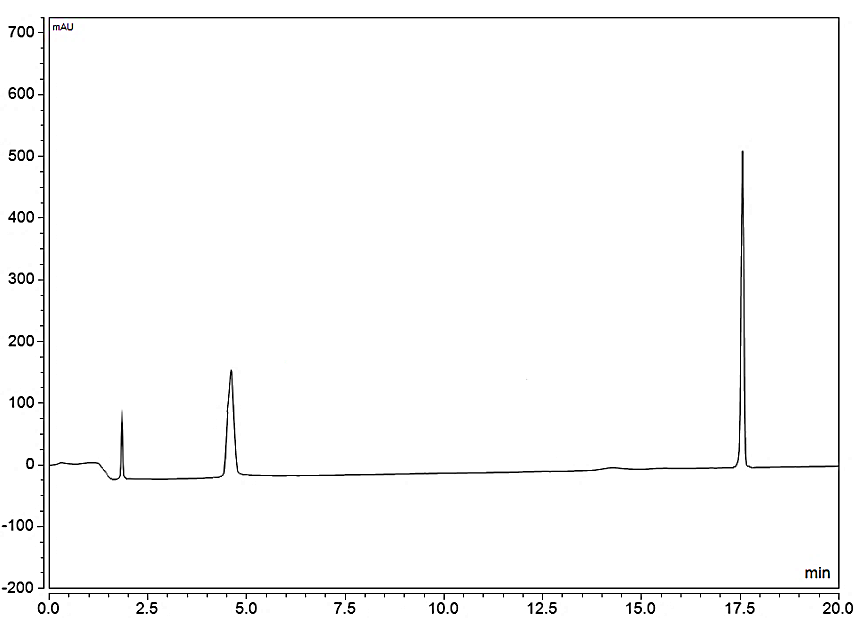

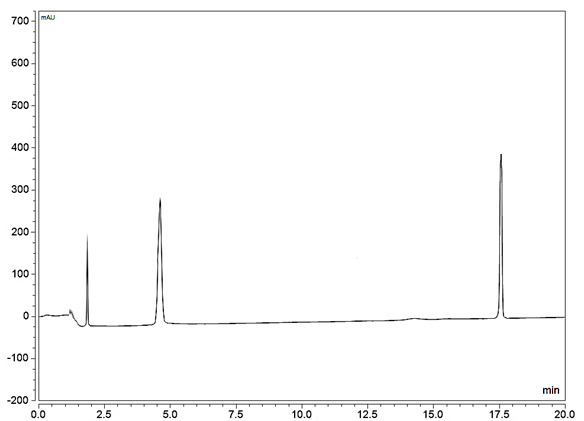
**

**DP_4_**

**4.82 min**

**DP_4_**

**4.82 min**

**DP_3_**

**1.98 min**

**1.98 min**

**DP_3_**

**1.98 min**

**b**

**a**

**
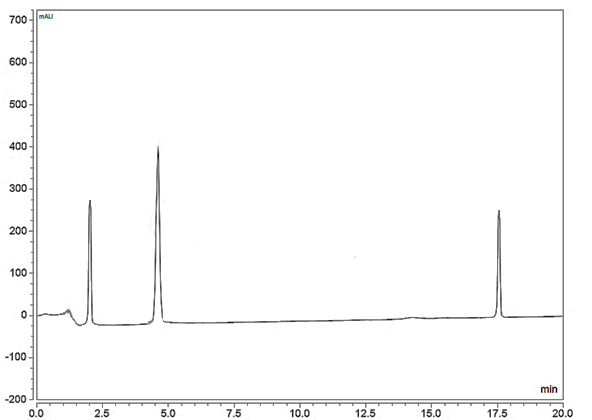
**

**c**

**
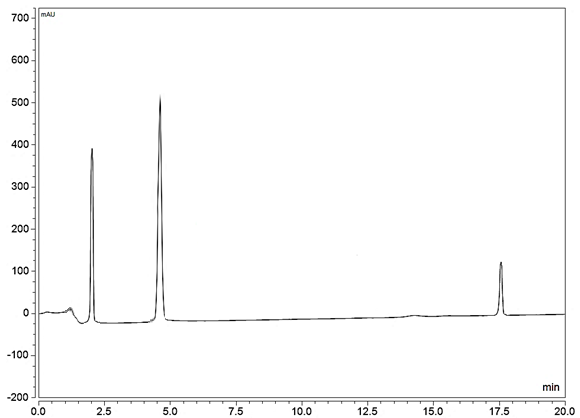
**

**d**

**DP_4_**

**4.82 min**

**DP_3_**

**1.98 min**

**DP_4_**

**4.82 min**

**DP_3_**

**1.98 min**

**Vilanterol**

**17.45 min**

**Vilanterol**

**17.45 min**

**
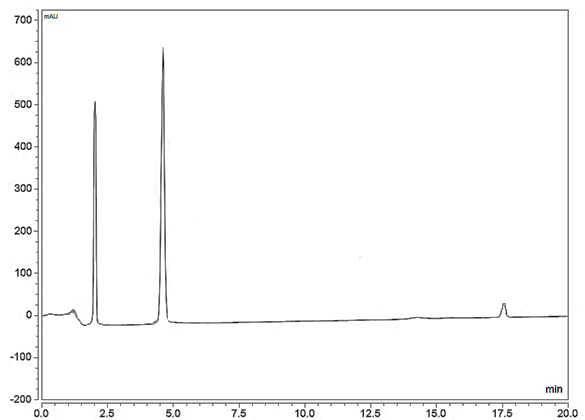
**

**e**

**DP_4_**

**4.82 min**

**DP_3_**

**1.98 min**

**Vilanterol**

**17.45 min**

**Fig. S4**

**
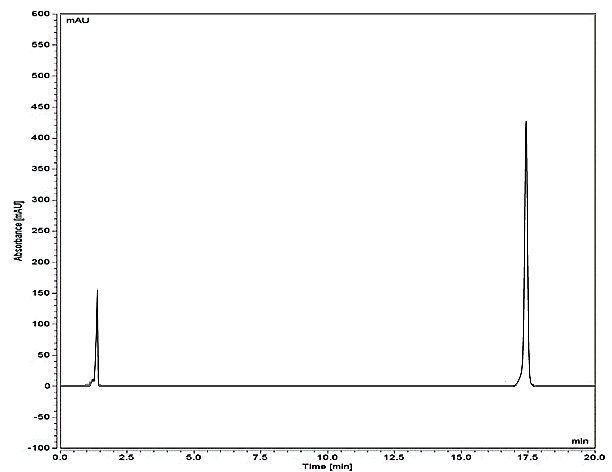

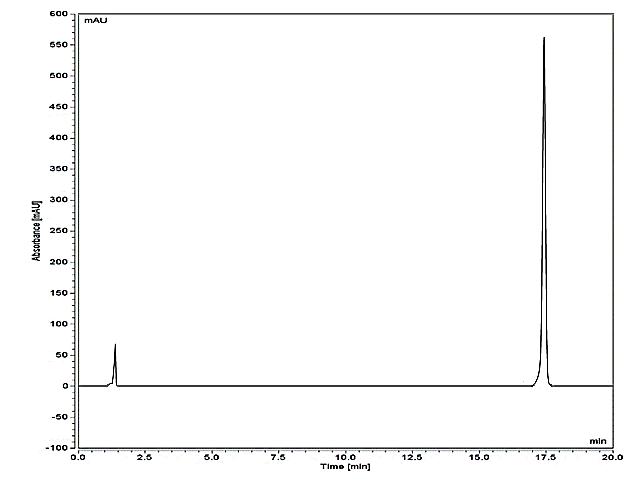
**

**DP_5_**

**1.48 min**

**DP_5_**

**1.48 min**

**Vilanterol**

**17.45 min**

**Vilanterol**

**17.45 min**

**b**

**a**

**
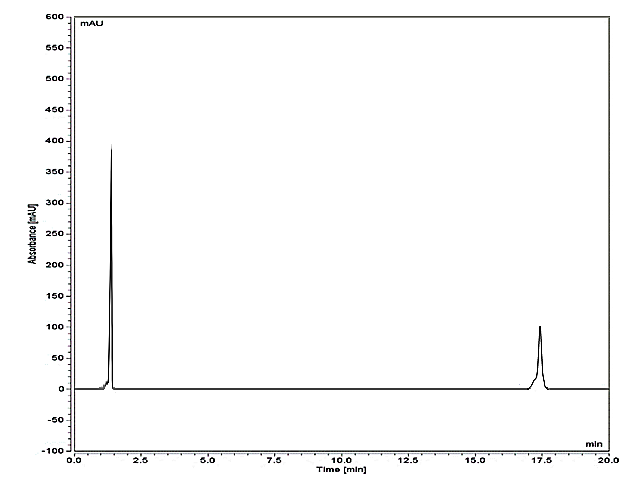

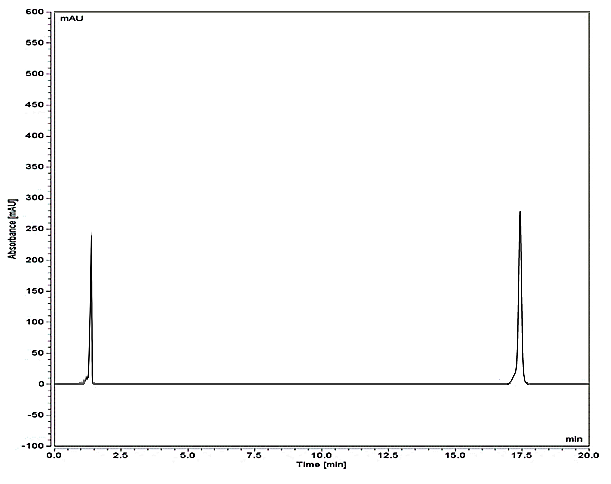
**

**d**

**c**

**DP_5_**

**1.48 min**

**DP_5_**

**1.48 min**

**Vilanterol**

**17.45 min**

**Vilanterol**

**17.45 min**

**
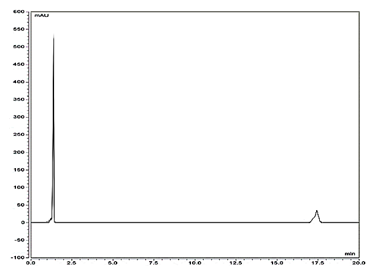

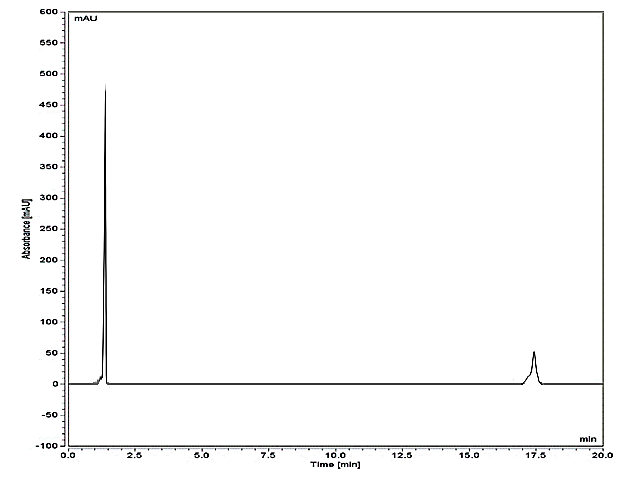
**

**e**

**DP_5_**

**1.48 min**

**DP_5_**

**1.48 min**

**f**

**Vilanterol**

**17.45 min**

**Vilanterol**

**17.45 min**

**Fig. S5**

**a**

**b**

**c**

**Fig. S6**

**References**

[1] Tiryaki, O., Özmen, D., Aydin, G. & Seçer, E. Setting System Suitability Parameters for Performance Optimization of GC-NPD Detection for Pesticide Residue Analysis. *J. Sci*. **22**, 149-155 (2009).

[2] Priyadarshini, G. I., Udayasri, N. N., Sowmya, M. L. & Pappula, N. Stability indicating RP-HPLC method development and validation for the simultaneous determination of vilanterol trifinatate and umeclidinium bromide in bulk and pharmaceutical dosage forms. *World j. pharm. pharm. sci.* **9**, 137-143 (2021).

[3] Hamsa, A. et al. Development and Validation of Stability Indicating Reverse Phase High Performace Liquid Chromatography Method for the Determination of Umeclidinium and Vilanterol in Pharmaceutical Dosage Form. *Int. J. Pharm. Pharm. Res*. **33**, 208-219 (2021). <https://doi.org/10.9734/jpri/2021/v33i42A32398>.

[4] Sindhu, D. S. & Rani, S. S. Stability indicating RP-HPLC method development and validation for the simultaneous estimation of vilanterol and umeclidinium bromide in bulk and pharmaceutical dosage forms. *World j. pharm. pharm. sci.* **10**, 128-135 (2022). <https://doi.org/10.54037/WJPS.2022.100114>.
